# Impact of COVID-19 Vaccination on General Surgical Emergencies in Al-Qassim Region, Saudi Arabia: A Single-Center Retrospective Chart Review

**DOI:** 10.7759/cureus.43630

**Published:** 2023-08-17

**Authors:** Hayfa Alolayan, Moath Aljohani, Mohammed Alfehaid, Ghadi AlMatroudi, Noura AlDhowyan, Joud AlQathlan, Shoug AlSuhaibani, Tahani AlShamikh, Ghadeer AlJohani, Anwaar AlSalamah, Hanadi AlRashidi

**Affiliations:** 1 Department of Surgery, Unaizah College of Medicine and Medical Sciences, Qassim University, Unaizah, SAU; 2 Department of Family and Community Medicine, Unaizah College of Medicine and Medical Sciences, Qassim University, Unaizah, SAU; 3 Department of Medicine, Unaizah College of Medicine and Medical Sciences, Qassim University, Unaizah, SAU

**Keywords:** appendicitis, pandemic, vaccine, covid-19, surgical emergencies

## Abstract

Background

This study sought to determine the COVID-19 pandemic and vaccination’s effects on the number of patients presenting with emergent surgical illnesses or requiring emergency general surgical procedures. We compared the number of presenting cases and surgical emergencies before the pandemic, in 2019, and during the pandemic, before and after the COVID-19 vaccination’s introduction.

Method

This observational retrospective chart review was conducted at a tertiary hospital in Al-Qassim, Saudi Arabia. The data were retrospectively collected for three periods (July 1 to September 30) in 2019, 2020, and 2021 using a data collection sheet for demographic data, visit date, comorbidities, emergency procedure type, COVID-19 test result, length of hospitalization, ICU admission status, and surgical case mortality.

Results

The study included 152 participants with a mean age of 36.1 (SD: 16) years, and 69.7% of them were male. Common surgical conditions were identified as acute appendicitis (49.3%), skin abscesses and pilonidal sinus (21.7%), and diabetic foot (9.2%) across all three years. The most frequent (48.7%) surgical procedure was appendectomy. A decrease in surgical emergencies rate was observed during the year 2020, as compared to 2019 and 2021. The general surgery emergency rate was highest in 2021 among patients admitted for procedures post-vaccination (38.8%).

Conclusion

Common surgical emergencies were most frequent in 2021, after the COVID-19 vaccine’s introduction. Future research areas include the impact of the pandemic on delays or the severity and complication of surgical or medical cases.

## Introduction

COVID-19 is a contagious respiratory disease characterized by respiratory symptoms, including difficulty breathing, fever, and dry cough [1]. WHO announced the virus as a pandemic on March 11, 2020 [1]. The first case of COVID-19 in the Kingdom of Saudi Arabia (KSA) was reported on March 2, 2020 [2].

The COVID-19 pandemic has significantly affected hospital surgical departments in several ways. For example, emergency surgeries have significantly decreased, while mortality in patients undergoing surgeries has significantly increased alongside the cancellation of elective cases, delays in surgical interventions, and closure of some private surgical practices due to financial burdens [3-11]. One of the biggest challenges this virus presents in the operating room is that the viral load in various bodily compartments and fluids is unknown, causing healthcare workers to operate in an unpredictable and dangerous environment [3,12]. In addition, published guidelines have issued varying recommendations, including that electrocautery should be done under minimum power settings and to avoid lengthy dissection in one area to reduce exposure to particles generated during surgeries. In addition, any needle-stick injuries or personal protective equipment (PPE) damage during operations must receive more attention in a pandemic environment [13].

Governments and non-governmental organizations worldwide have introduced measures to stop or slow the spread of COVID-19 [4-6]. For example, the Royal Colleges of Surgeons in Ireland and Great Britain have recommended a strategy for emergency surgery settings: using PPE, employing conservative management whenever suitable, and using open rather than laparoscopic operations unless the laparoscopic approach offers significant clinical benefits to the patient [14]. Moreover, the COVID-19 pandemic’s effect on surgical emergencies has been prominent internationally. One study across 24 countries included 1128 patients who had surgery between January 1 and March 31, 2020, of whom 74.0% had emergency surgery and 24.8% had elective surgery; it found that the 30-day mortality percentage was 23.8%, a higher proportion of which occurred postoperatively in emergency surgery (rather than in elective surgery) [7]. Males experienced higher mortality rates than females, as did the older population (70 years old and older), and people with pulmonary complications constituted 82.6% of all deaths [7].

In KSA, similar to other countries, surgical beds have been nationally redistributed to care for COVID-19 admissions. Surgical bed capacity reductions and an increased risk of infection among surgical patients were concerning [4-6,8]. Health authorities limited surgical visits in outpatient clinics and wards admission to protect patients and healthcare staff. According to the Ministry of Health (MOH), as of February 21, 2020, elective surgeries were delayed, with some exceptions, while other urgent cases have also been rescheduled [8]. The KSA was among the first countries to introduce COVID-19 vaccines for its citizens to prevent and reduce the spread of the infection and the severity of the disease. The first vaccination campaign began on December 17, 2020. On July 11, 2021, the MOH announced that 50% of the population and residents in KSA had been immunized with the first dose of the COVID-19 vaccine. The vaccine’s introduction in KSA has positively contributed to its emergency surgical department [15].

The impact of COVID-19 vaccination on surgical emergencies in KSA has not been directly addressed in the available literature. However, the research do provide information on several aspects related to COVID-19 in KSA. One study reported factors affecting COVID-19 vaccine hesitancy among the general population in KSA [16]. Another report described the characteristics and pattern of emergency department visits during the COVID-19 pandemic, including demographic features, acuity level, length of stay, and admission rate [17]. Moreover, there was a review of urologists’ response to the COVID-19 pandemic in KSA, including the implementation of measures to minimize outpatient clinics and the use of telemedicine [18]. Additionally, an investigation examined the impact of lockdown on injuries during the pandemic in a level-I trauma center in Riyadh, KSA [19]. There is also research on the impact of COVID-19 on healthcare systems, including the postponement of elective surgeries [20]. Therefore, in this study, we aim to determine and compare the rate of surgical emergencies before and during the pandemic and after the introduction of COVID-19 vaccines in KSA. The findings of this study would lead the way to develop management guidelines for surgical emergencies, recommend vaccination policies for surgical patients in case of future similar situations, or identify new areas of research related to COVID-19 infection.

## Materials and methods

Study design, setting, and procedure

The study was a quantitative observational retrospective cross-sectional study. It was conducted at the tertiary hospital in Unaizah, Al-Qassim, KSA, the only governmental care hospital in the city, with a capacity of 360 beds. We collected the data retrospectively from surgical emergency records in this tertiary facility. The selection period was from July 1 to September 30 for 2019, 2020, and 2021. The years selected represent the stages before and during the pandemic as well as after the COVID-19 vaccine’s introduction in KSA. This study included all surgical emergencies admitted during the study period and excluded all non-general surgical cases and elective general surgeries. The patients who refused to undergo their surgeries and signed for discharge against medical advice due to the fear of getting infected were also excluded. All patients meeting the inclusion criteria from all selected study periods were included in our analysis. We conducted a comprehensive review of medical records on specific variables included in our structured data collection sheet and data were compared between the selected periods of three years.

This study was registered at http://www.researchregistry.com and reported according to STROCSS criteria [21]. All the procedures performed in this study aligned with the ethical standards and were approved (reference no: 1443-3-5453, dated September 30, 2021) by the regional ethical committee, and the consent is waived due to the nature of the study design. In addition, approval was obtained from the hospital’s administration and department head. All retrieved data was used without any personal identifiers to protect the anonymity and confidentiality of the medical records.

Study tool

Data were collected using a structured data collection sheet based on the review of previous studies [3,22]. In addition, three experts in the field ensured the data collection sheet’s validity. Information was obtained from medical records, including demographic data (age, gender), visit date, admission diagnosis, and presence of comorbidities including diabetes, hypertension, coronary artery diseases, chronic kidney diseases, and others. Data related to the length of hospitalization, COVID-19 test results for 2020 and 2021, type of emergency procedures, ICU admissions of surgical cases, and hospital mortality of surgical cases were also included to achieve the study aim. A pilot study included 17 records was conducted to test the feasibility, reliability, and time needed to complete and the clarity of the data collection sheet before conducting the study. The sample size estimation was based on including all cases that fit inclusion criteria for a period of three months in each year from 2019 to 2021 to reach a sufficient sample size. Furthermore, another assumption was taken into account which is calculated through the assumption of 10 cases per variable as per Sackett et al. [23], which accounts for 100 cases, and then we added 10 cases to account for the incompleteness of data totaling up to 110 cases.

Statistical analysis

Data collected from the participants were entered into a Microsoft Excel spreadsheet (Microsoft, Washington, USA), where it was reviewed, validated, cleaned, and prepared for analysis. Analyses were completed using SPSS Statistics version 22 (IBM Corp. Released 2013. IBM SPSS Statistics for Windows, Version 22.0. Armonk, NY: IBM Corp.). The authors completed descriptive analyses to assess the distribution of the participants across the three periods used for the study: July to September 2019, July to September 2020, and July to September 2021. The results of descriptive analyses were presented in a frequency/proportion table. In addition, trends concerning the monthly and weekly rates of general surgical emergencies during the three years were determined, with the results presented in charts. Inferential analyses were conducted using chi-square tests (for categorical variables) and the Kruskal-Wallis test (for continuous variables, such as age) to determine the presence and magnitude of any associations between selected variables and the rate of general surgical emergencies in the three periods. The chi-square test compares the observed frequencies of each group to the expected frequencies under the null hypothesis. The Kruskal-Wallis test ranks the data of all groups together and then compares the mean ranks of the different groups. The Kolmogorov-Smirnov test was implemented to determine the degree of normality in the distribution of continuous variables, such as age and hospitalization length. The level of significance was set at p <0.05.

## Results

General characteristics and distribution of the entire study population

The study included 152 participants who met the inclusion criteria, with a mean age of 36.1 (SD: 16.0) years (Table 1). Male-to-female, the sex of the participations was skewed at 69.7% to 30.3%. All participants had been admitted for a wide range of surgical conditions, with the most common being acute appendicitis (49.3%), followed by skin/subcutaneous abscess (including perianal region, gluteal region, abdomen, thigh, neck, chest/breast, upper back, upper arm, and hand abscess), pilonidal sinus (21.7%), and diabetic foot (9.2%). For their management, different general surgical procedures were implemented. A considerable number of patients had an appendectomy (48.7%), and another 21.7% had irrigation and drainage for skin and subcutaneous abscesses. For those that had diabetic foot ulcers, there was diabetic foot debridement or amputation (8.6%).

**Table 1:** General characteristics and distribution of the study population SD: standard deviation, ICU: intensive care unit, I&D: incision and drainage

Variables	Values	Frequency	%
Age, mean ± SD		36.1 ± 16.0	
Sex	Male	106	69.7
	Female	46	30.3
Diagnosis	Acute appendicitis	75	49.3
	Skin/subcutaneous abscess/pilonidal sinus	33	21.7
	Diabetic foot	14	9.2
	Obstruction, regardless of the cause	8	5.3
	Perforation (gastric/bowel)	6	3.9
	Hernia (all)	6	3.9
	Cholecystitis/pancreatitis	4	2.6
	Trauma	2	1.3
	Anorectal disease	1	0.7
	Others	3	2.0
COVID-19 testing	Negative	23	15.1
	Positive	2	1.3
	Result missing	1	0.7
	Not requested or applicable	126	82.9
Procedures	Appendectomy	74	48.7
	I&D for skin and subcutaneous abscess	33	21.7
	Diabetic foot debridement and/or amputation	13	8.6
	Hernia repair with/without bowel resection	6	3.9
	Adhesiolysis	5	3.3
	Bowel resection	4	2.6
	Repair of bowel perforation	4	2.6
	Cholecystectomy (both open and laparoscopic)	4	2.6
	Others	9	5.9
ICU admission	Yes	19	12.5
	No	133	87.5
Mortality	Yes	1	0.7
	No	151	99.3
Hypertension	Yes	22	14.5
	No	130	85.5
Diabetes mellitus	Yes	34	22.4
	No	118	77.6
Coronary artery disease	Yes	12	7.9
	No	140	92.1
Chronic kidney disease	Yes	5	3.3
	No	147	96.7
Other chronic diseases	Yes	14	9.2
	No	138	90.8

As part of care management, only about 17% of the population were tested for the COVID-19 virus, among which only two (7.7%) patients had confirmed positive results. Nineteen participants (12.5%) required care in the ICU, and among the entire population, one patient died within the study period. Regarding comorbidities, diabetes mellitus was the most common comorbidity encountered (22.4%), followed by hypertension (14.5%) and coronary artery disease (7.9%).

Trends in hospital admissions

**Figure 1:**
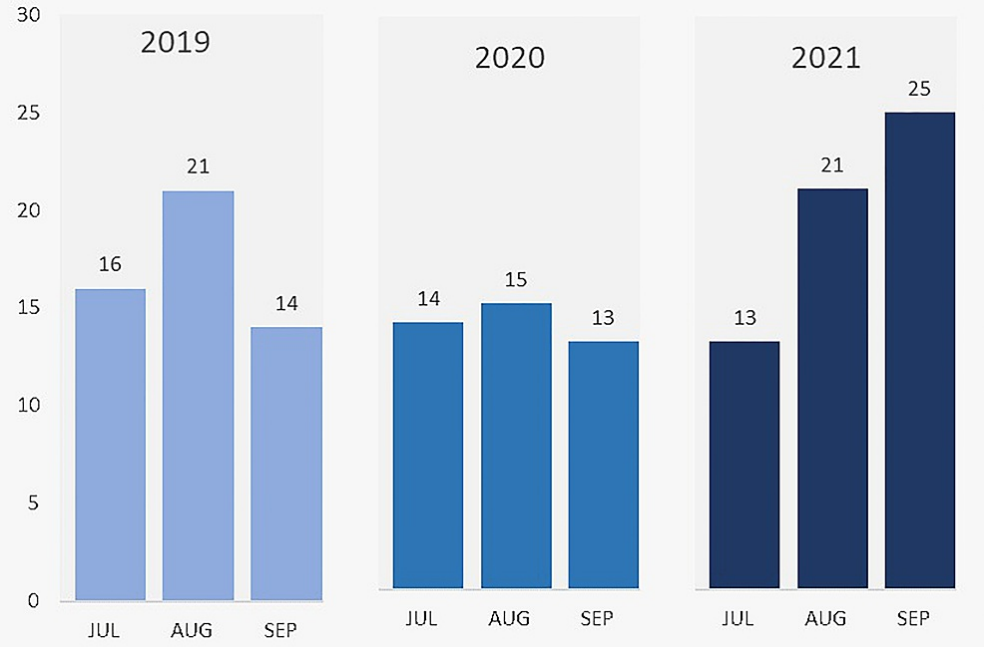
Monthly trends for hospital admissions between July and September of 2019, 2020, and 2021 in bar graph form. The x-axis represents the month and year of admission, and the y-axis represents the number of admitted patients

**Figure 2:**
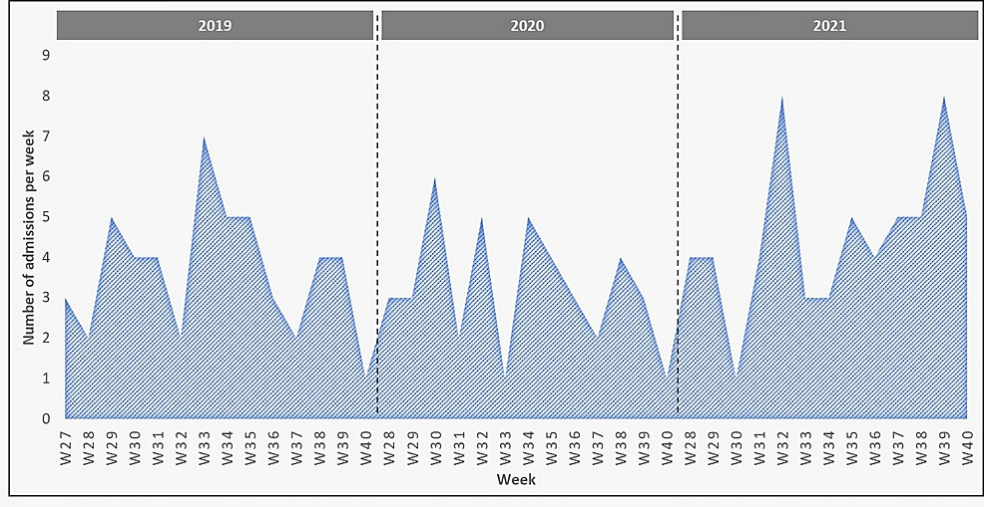
Weekly admission trends in 2019, 2020, and 2021. The x-axis represents the week and year of admission, and the y-axis represents the number of admitted patients per week

Across the entire population, the rate of general surgery emergencies was highest in 2021, with 59 patients admitted for various procedures during this period (38.8%). The year 2019 had the second-largest group of patients admitted for general surgery procedures (n = 51, 33.6%). Looking at the monthly trends during each year, the rate of general surgery emergencies was highest during August 2019, August 2021, and September 2021 (Figure 1). Additionally, the weekly average number of emergencies in 2019 was 3.6 (SD 1.6, 95% CI 2.7-4.6). This reduced by 11.3% to 3.2 (SD 1.5, 95% CI 2-3-4.2) cases per week in 2020. However, the weekly average increased from 3.2 to 4.54 (SD 1.9, 95% CI 3.4-5.7) cases per week, an increase of 40.6% from the previous year (Figure 2).

Associations between selected variables and rate of general surgical emergencies between 2019 and 2021

Additional analyses were completed to determine relationships between selected variables and the rate of surgeries during the three periods studied (Table 2). Patient ages across the three years were generally similar. It was highest in 2019 (38.8 ± 17.7 years) and lowest in 2021 (34.5 ± 14.8), but the difference was not found to be statistically significant (p =0.321). The diagnoses were also generally similar across the three years, with acute appendicitis as predominant-45.1% in 2019 to 54.2% in 2021 (p =0.790). Diagnoses of skin or subcutaneous abscesses or pilonidal sinus came in second place during the three periods, with proportions ranging from 19.0% to 25.4%. Aligned with the spread of diagnoses between 2019 and 2021, the types of surgical procedures carried out followed a similar trend, with appendectomy as the most-performed procedure in each of the periods, ranging from 45.1% in 2019 to 52.5% in 2021; however, the differences were not statistically significant (p = 0.576).

Regarding COVID-19 test results, most COVID-19 tests were conducted in 2021. None were implemented in 2019, before the beginning of the pandemic. In 2021, 25.4% of the patients admitted that year required ICU admission, the highest across the three-year range of this study (p =0.001), indicating a significantly higher proportion of patients were admitted to the ICU in 2021. Only 2.4% of the 41 patients seen in 2020 received ICU care. The length of hospitalization (including both pre- and postoperative periods) showed no statistically significant difference between the number of days patients spent on in-hospital care across the three years (p =0.190). On average, patients spent the longest time hospitalized in 2019 (6.8 ± 11.8 days) and the shortest time in 2020 (3.9 ± 4.0 days). The prevalence rates of comorbidities such as hypertension, diabetes mellitus, and coronary artery disease were comparable across the three periods (p >0.05).

**Table 2:** Comparison of patient characteristics and outcomes pre-pandemic, during, and post-pandemic * Kruskal-Wallis test carried out based on the non-normal distribution of data ** No COVID-19 testing was done in 2019 Bold: significant difference at a p-value below 0.05

Variables	2019 (n = 51) N (%)	2020 (n = 42) N (%)	2021 (n = 59) N (%)	p-value
Age, median (IQR)	35.0 (31.0)	31.0 (25.0)	33.0 (22.0)	0.461*
Mean ± SD	38.8 ± 17.7	35.0 ± 15.2	34.5 ± 14.8	
Gender				
Male	35 (68.6%)	33 (78.6%)	38 (64.4%)	0.305
Female	16 (31.4%)	9 (21.4%)	21 (35.6%)	
Diagnosis				
Acute appendicitis	23 (45.1%)	20 (47.6%)	32 (54.2%)	0.790
Skin/subcutaneous abscess/pilonidal sinus	10 (19.6%)	8 (19.0%)	15 (25.4%)	
Diabetic foot	4 (7.8%)	6 (14.3%)	4 (6.8%)	
Obstruction, regardless of the cause	5 (9.8%)	1 (2.4%)	2 (3.4%)	
Perforation (gastric/bowel)	2 (3.9%)	1 (2.4%)	3 (5.1%)	
Hernia (all)	2 (3.9%)	2 (4.8%)	2 (3.4%)	
Trauma	1 (2.0%)	1 (2.4%)	0 (0.0%)	
Cholecystitis/pancreatitis	2 (3.9%)	1 (2.4%)	1 (1.7%)	
Anorectal disease	1 (2.0%)	0 (0.0%)	0 (0.0%)	
Others	1 (2.0%)	2 (4.8%)	0 (0.0%)	
COVID-19 test**				
Negative	0 (0.0%)	7 (16.7%)	16 (27.1%)	0.001
Positive	0 (0.0%)	2 (4.8%)	0 (0.0%)	
Result missing	0 (0.0%)	1 (2.4%)	0 (0.0%)	
Not requested or applicable	51 (100.0%)	32 (76.2%)	43 (72.9%)	
Procedure				
Appendectomy	23 (45.1%)	20 (47.6%)	31 (52.5%)	0.576
I&D for skin and subcutaneous abscess	10 (19.6%)	8 (19.0%)	15 (25.4%)	
Diabetic foot debridement and/or amputation	4 (7.8%)	5 (11.9%)	4 (6.8%)	
Adhesiolysis	5 (9.8%)	0 (0.0%)	0 (0.0%)	
Hernia repair with/without bowel resection	2 (3.9%)	2 (4.8%)	2 (3.4%)	
Bowel resection	1 (2.0%)	1 (2.4%)	2 (3.4%)	
Repair of bowel perforation	1 (2.0%)	1 (2.4%)	2 (3.4%)	
Cholecystectomy (both open and laparoscopic)	2 (3.9%)	1 (2.4%)	1 (1.7%)	
Others	3 (5.9%)	4 (9.5%)	2 (3.4%)	
ICU admission				
Yes	3 (5.9%)	1 (2.4%)	15 (25.4%)	0.001
No	48 (94.1%)	41 (97.6%)	44 (74.6%)	
Length of hospitalization, median (IQR)	3.0 (5.0)	2.0 (4.0)	2.0 (3.0)	0.586*
Mean ± SD	6.8 ± 11.8	3.9 ± 4.0	4.6 ± 5.9	
Mortality				
Yes	0 (0.0%)	0 (0.0%)	1 (1.7%)	0.452
No	51 (100.0%)	42 (100.0%)	58 (98.3%)	
Hypertension	8 (15.7%)	6 (14.3%)	8 (13.6%)	0.950
Diabetes mellitus	13 (25.5%)	9 (21.4%)	12 (20.3%)	0.800
Coronary artery disease	3 (5.9%)	1 (2.4%)	8 (13.6%)	0.098
Chronic kidney disease	2 (3.9%)	1 (2.4%)	2 (3.4%)	0.916
Other chronic conditions	6 (11.8%)	4 (9.5%)	4 (6.8%)	0.664

## Discussion

With the number of patients admitted at 51 and 42 in 2019 and 2020, respectively, the rate of general emergencies decreased in 2020 when compared to 2019. After the COVID-19 vaccine’s introduction, this reduction was followed by an increase in the rate in 2021, with 59 patients admitted for general surgical emergencies. Our results are consistent with a previous study in which there was a reduction (59.1%) in surgical emergencies [24]; furthermore, it is reported that during the pandemic, the consultation rate for general surgery emergencies decreased significantly from 195 patients before the pandemic to 132 patients after it [25], a 54% reduction in emergency surgeries in India, while a study from Italy documented 86% decline in emergency surgery in the first month after nation-wide lockdown [26-27]. Likewise, a 70% downturn in surgical admissions and a 50% drop in surgeries were observed during the Ebola pandemic in Sierra Leone [28]. This marked reduction in emergency surgical procedures is also supported by several studies worldwide [29-35]. The decrease in emergency operations could plausibly be attributed to a diversion of resources to the management of patients with COVID-19, as well as a lockdown resulting in suboptimal healthcare facilities access [36-38]. Moreover, fear of contracting COVID-19 may have also been a contributing factor [39].

The finding in Table 2 highlights the significant decrease in female patients during the pandemic. Females accounted for 31.4% in 2019 and 35.6% in 2021 but 21.4% during the pandemic, similar to the findings of a study by Tarim et al. [25]. This finding could potentially result from women heeding pandemic warnings more than men, women refraining from visiting the emergency room, or women overusing emergency services before the outbreak [25].

Across the three years of our study, patients’ diagnoses were similar, with no significant difference. Acute appendicitis was the most common diagnosis, ranging from 45.1% in 2019 to 54.2% in 2021. Similarly, the types of procedures performed followed the same pattern in all years, with appendectomies being the most performed, ranging from 45.1% in 2019 to 52.5% in 2021. However, previous studies found a decrease in the number of cases admitted with acute appendicitis and the number of appendectomies performed [3,24] This discrepancy might be due to using non-operative approaches, such as prescribing antibiotics [24]. No significant difference was found in the number of trauma cases admitted within general surgical emergencies. Similar previous studies also compared the pandemic’s effect on the rate of trauma cases, finding that there was no significant difference in trauma cases admitted before and after the pandemic [3,24-25].

According to our study, COVID-19 testing was most common in 2021. However, in the year 2020, only two patients were positive. In addition, as COVID-19 testing was not requested for most cases or the result was missed during 2020 and 2021, this limits the study’s broader applicability and might affect comparisons. In a review by another study, the authors recommended that all patients undergoing surgery should be tested for COVID-19 without delay to reduce the risk of transmission of COVID-19 [40]. In contrast, a Scottish study reported that most emergency general surgical patients did not undergo COVID-19 testing due to concerns over sensitivity and testing costs during the study period [41].

Regarding ICU admission, the highest rate across the three years of this study was observed in 2021, when 25.4% of patients admitted that year required ICU admission. However, only 5.9% of the 48 patients in 2019 and 2.4% of the 41 patients seen in 2020 were managed in the ICU. The present study’s results for ICU admission pre-pandemic and during the pandemic aligned with another study’s results [42]. This finding may be explained by how, during the period of post-vaccine release, the MOH stipulated that entry to all buildings affiliated with the Ministry would be restricted based on immunization status.

Upon examining the length of hospitalization (including pre- and postoperative periods), patients spent the longest time on average in 2019 (6.8 ± 11.8 days) and the shortest time in 2020 (3.9 ± 4.0 days). However, there was no statistically significant difference between the number of days patients spent on in-hospital care across the three years. Our results align with a study done in Spain [43] and may be due to early discharge from the hospital during the COVID-19 pandemic due to the restrictions that aim to limit the spread of infection and expand the hospital beds’ capacity [44].

Regarding comorbidities, diabetes mellitus was the most common comorbidity encountered (22.4%), followed by hypertension (14.5%) and coronary artery disease (7.9%). Although there were no significant differences in the prevalence rate of comorbidities in all three time periods, this finding aligns with a study conducted by Surek et al. [24] However, another study showed an increase in morbidity rate during the pandemic time [25,42].

In this study, the mortality rate of patients undergoing emergency surgeries did not show a statistically significant difference across the three studied periods. This finding aligned with another study [43] reporting that the mortality and reoperation rates were similar in the control and pandemic groups. A previous study reported that the time between the onset of complaints and admission to the emergency room during the pre-pandemic period has not changed during the pandemic [25]. However, this area was unaddressed in our study; future studies could explore this area by measuring the impact of the pandemic and relevant restrictions on the delays of case presentation and subsequent increases in the rate and severity of complications, even for surgical and medical cases.

We encountered several limitations in conducting our research. First, the sample size was small and limited to one city from one local hospital, the only governmental care hospital in the city, and does not include the whole months of the year which may affect the generalizability of the findings and may not capture the comprehensive regional or national trends in surgical emergencies during the COVID-19 pandemic and its vaccination period. Additionally, postoperative ICU admissions and postoperative complications like nosocomial infection and paralytic ileus must also be studied. Furthermore, the COVID-19 swab results were missed or not requested for some patients, and the timeframe from the onset of the complaints until seeking medical care was not addressed as these data were absent from most patients’ files. Therefore, this absence of COVID-19 test results for some patients led to our inability to analyze the impact of COVID-19 infections on surgical emergencies during selected periods. Nevertheless, this study is sought to describe the impact of COVID-19 and its vaccine on emergency surgeries in our region.

## Conclusions

The incidence of common surgical emergencies was highest in 2021, after the COVID-19 vaccine’s introduction, with most patients presenting with acute appendicitis, skin or subcutaneous abscesses (including perianal region, gluteal region, thigh, abdomen, neck, chest/breast, upper back, upper arm, and hand abscess), or diabetic feet; they underwent surgical procedures to manage these cases. Future research utilizing larger and more heterogeneous samples, including multiple healthcare centers, would be helpful in enhancing the generalizability of the findings. Moreover, future studies could examine additional variables, such as the pandemic’s impact on the delays in case presentations and the increased rate or severity of the complications, to achieve a more comprehensive understanding of the pandemic's influence on surgical care. Consequently, there is a need for further research to explore the complete implications of the pandemic and vaccination on surgical care.
